# Personalized predictions of neoadjuvant chemotherapy response in breast cancer using machine learning and full-field digital mammography radiomics

**DOI:** 10.3389/fmed.2025.1582560

**Published:** 2025-04-17

**Authors:** Ye Ruan, Xingyuan Liu, Yantong Jin, Mingming Zhao, Xingda Zhang, Xiaoying Cheng, Yang Wang, Siwei Cao, Menglu Yan, Jianing Cai, Mengru Li, Bo Gao

**Affiliations:** ^1^Department of Radiology, The Second Affiliated Hospital of Harbin Medical University, Harbin, China; ^2^Department of Breast Surgery, Harbin Medical University Cancer Hospital, Harbin, China

**Keywords:** breast cancer, radiomics, neoadjuvant chemotherapy, machine learning, full-field digital mammography, nomogram

## Abstract

**Objective:**

This study aimed to develop a comprehensive nomogram model by integrating clinical pathological and full-field digital mammography (FFDM) radiomic features to predict the efficacy of neoadjuvant chemotherapy (NAC) in breast cancer patients, thereby providing personalized treatment recommendations.

**Methods:**

A retrospective analysis was conducted on the clinical and imaging data of 227 breast cancer patients from 2016 to 2024 at the Second Affiliated Hospital of Harbin Medical University. The patients were divided into a training set (*n* = 159) and a test set (*n* = 68) with a 7:3 ratio. The region of interest (ROI) was manually segmented on FFDM images, and features were extracted and gradually selected. The rad-score was calculated for each patient. Five machine learning classifiers were used to build radiomics models, and the optimal model was selected. Univariate and multivariate regression analyses were performed to identify independent risk factors for predicting the efficacy of NAC in breast cancer patients. A nomogram prediction model was further developed by combining the independent risk factors and rad-score, and probability-based stratification was applied. An independent cohort was collected from an external hospital to evaluate the performance of the model.

**Results:**

The radiomics model based on support vector machine (SVM) demonstrated the best predictive performance. FFDM tumor density and HER-2 status were identified as independent risk factors for achieving pathologic complete response (PCR) after NAC (*p* < 0.05). The nomogram prediction model, developed by combining the independent risk factors and rad-score, outperformed other models, with areas under the curve (AUC) of 0.91 and 0.85 for the training and test sets, respectively. Based on the optimal cutoff points of 103.42 from the nomogram model, patients were classified into high-probability and low-probability groups. When the nomogram model was applied to an independent cohort of 47 patients, only four patients had incorrect diagnoses. The nomogram model demonstrated stable and accurate predictive performance.

**Conclusion:**

The nomogram prediction model, developed by integrating clinical pathological and radiomic features, demonstrated significant performance in predicting the efficacy of NAC in breast cancer, providing valuable reference for clinical personalized prediction planning.

## Introduction

Breast cancer has the highest incidence rate of cancer in women (1). As a systematic treatment method, neoadjuvant chemotherapy (NAC) has been widely used in patients presenting with locally advanced tumors or in those for whom surgical intervention is indicated (2, 3). It can detect tumor sensitivity to the drug, reduce the local size of the tumor, improve the breast-conserving surgery rate (4) and even achieve pathological complete response (PCR). PCR is defined as the absence of residual invasive carcinoma in both the primary tumor bed [ductal carcinoma *in situ* (DCIS) may persist] and regional lymph nodes (including ipsilateral sentinel and axillary nodes) (5). Patients who achieve PCR usually have longer disease-free survival, overall survival and a lower risk of recurrence, with significantly improved patient survival rates (6). However, only a fraction of breast cancer patients can achieve PCR after NAC treatment (7). Some patients experience adverse outcomes due to chemotherapy toxicity and associated adverse effects, which may unexpectedly lead to accelerated disease progression (8). Therefore, accurate prediction of efficacy before neoadjuvant chemotherapy is crucial for optimizing patient outcomes. Currently, the evaluation of NAC efficacy is predominantly conducted through pathological and clinical assessments. Pathological evaluation is widely regarded as the gold standard for assessing NAC efficacy; however, being retrospective, it cannot predict therapeutic outcomes in advance. The primary clinical evaluation techniques encompass Full-field digital mammography (FFDM), ultrasonography, and magnetic resonance imaging (MRI). In comparison to ultrasonography and MRI, FFDM offers the advantage of lower cost, simplicity in operation, and the ability to observe breast structure, abnormalities, and microcalcifications. Radiomics, as an emerging interdisciplinary field that combines imaging information and computer science to predict the treatment response and prognosis of patients through high-throughput extraction, quantitative analysis and mining of features in images (9). Radiomics has shown significant potential in recent years for enhancing the accuracy of breast cancer diagnosis, lymph node metastasis assessment, and prognosis prediction (10, 11). Machine learning is a method for constructing data-driven computational models that can enhance the performance and predictive capability of disease models, making it an essential component of radiomics (12). In the existing literatures, there is a preponderance of studies that combine MRI and ultrasound images with radiomics for predicting breast cancer response to NAC (13, 14). Even articles utilizing contrast-enhanced spectral mammography (CESM) images in conjunction with radiomics have emerged in an endless stream (15, 16). Regarding FFDM, Liu et al. (17) developed a deep learning model based on FFDM using Mask-RCNN to evaluate malignant architectural distortion, achieving good diagnostic performance. Zhang et al. (18) combined radiomics features and deep features to classify benign and malignant breast lesions on FFDM images, and the results indicated that this classification framework deonstrated excellent performance in the diagnosis of breast lesions. However, despite being the most widely used technique for breast cancer screening, its potential for radiomics-based prediction of NAC efficacy remains largely unexplored. The purpose of this study was to explore the value of clinical, machine learning, and radiomics nomogram models in predicting the efficacy of NAC for breast cancer.

## Materials and methods

### Patients

**Figure 1:**
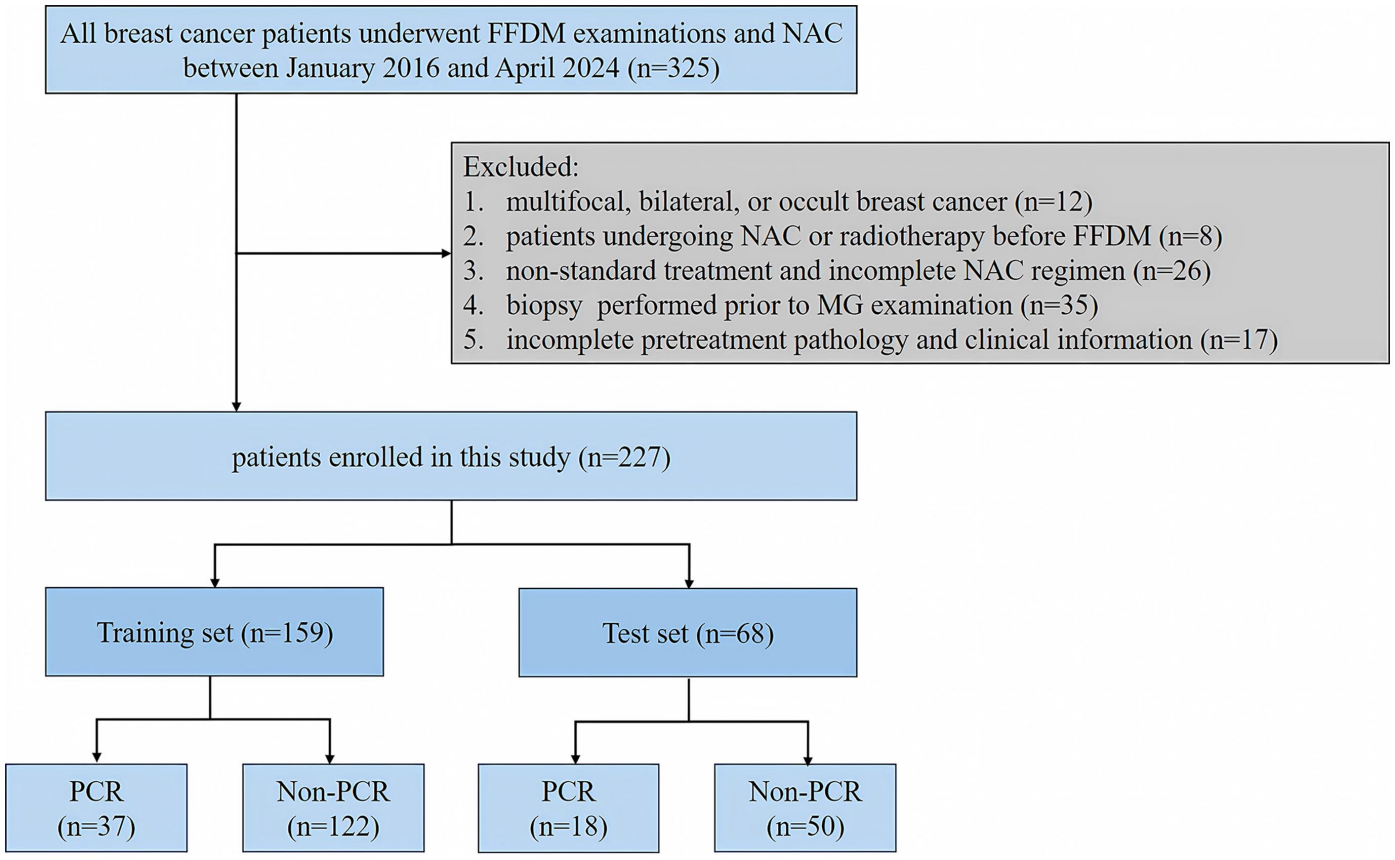
Flowchart of patients’ selection in this study.

A retrospective analysis of breast cancer patients who underwent NAC at the Second Affiliated Hospital of Harbin Medical University from January 2016 to April 2024. Inclusion criteria: (I) breast cancer patients with histologically confirmed diagnosis based on biopsy or resected specimens; (II) patients with mass-forming breast cancer; (III) patients who underwent pathological biopsy and surgery after NAC; (IV) patients with complete mammographic and clinicopathological data; Exclusion criteria: (I) multifocal, bilateral, or occult breast cancer; (II) patients undergoing NAC or radiotherapy before FFDM; (III) the patients who received non-standard treatment or did not complete the NAC regimen. (IV) patients who underwent biopsy prior to FFDM. We randomly assigned the finally enrolled 227 breast cancer patients into the training set and the test set in a 7:3 ratio. In the training set, 37 patients had PCR and 122 patients had non-PCR. In the test set, 18 patients were with PCR and 50 patients were with non-PCR. The flowchart of patient selection is shown in Figure 1.

### FFDM examination

In this study, breast imaging was acquired using the Hologic Lorad Selenia FFDM system to obtain craniocaudal (CC) and mediolateral oblique (MLO) views, with additional lateral views acquired as necessary. All FFDM images were independently diagnosed by two radiologists, each with over 10 years of experience in breast imaging diagnosis, with reference to the 5th edition of the Breast Imaging Reporting and Data System (BI-RADS) and without knowledge of the pathological results. In cases of disagreement, a consensus decision was reached through joint discussion.

### NAC assessment

According to the guidelines of the National Comprehensive Cancer Network (NCCN) and the American Society of Clinical Oncology (ASCO), the NAC regimens for breast cancer patients were based on anthracycline and taxane, while HER-2 positive patients received combination therapy with trastuzumab and pertuzumab. The NAC regimen primarily consisted of 6 to 8 courses (19, 20).

### Clinical and pathological characteristics

The baseline clinicopathological data include: patient age, BMI, clinical T stage, estrogen receptor (ER) status, progesterone receptor (PR) status, human epidermal growth factor receptor-2 (HER-2) status, and the Ki-67 proliferation index. Immunohistochemical (IHC) staining demonstrated ER/PR positivity when ≥1% of the tumor cells exhibit positive staining in their nuclei. The IHC results for HER-2 were deemed positive when (+++), negative when (+), and necessitates further evaluation by Fluorescence *In Situ* Hybridization (FISH) when (++). A FISH result showing amplification confirmed HER-2 positivity. Conversely, a non-amplified FISH result indicated HER-2 negativity. The cutoff level for Ki67 was 20%. The pathological response was assessed according to the Miller Payne criteria (21) as follows: Grade 1 indicates no reduction in tumor cell count; Grade 2, a reduction of <30% in tumor cells; Grade 3, a reduction of 30 to 90%; Grade 4, a reduction of >90%; and Grade 5, no infiltrative tumor cells observed in the tumor bed on histological sections.

### Region of interest segmentation and radiomics feature extraction from FFDM

Radiologist 1 (with 15 years of experience in breast imaging) utilized the 3D Slicer software to annotate and crop the region of interest (ROI) where the tumor was located on the FFDM grayscale images. Then, the second radiologist (with 12 years of experience in breast imaging) segmented the ROIs for images from 60 randomly selected patients. After segmenting the ROIs for all FFDM images, we utilized the RIAS MIT V0.2 software to extract features, resulting in a suite of radiomic features that includes 190 first-order statistical features, 750 texture features, 34 shape features, and 1,504 wavelet features. The texture features included 240 gray co-occurrence matrix features (GLCM), 140 Gy level dependence matrix features (GLDM), 160 Gy level run length matrix features (GLRLM), 160 Gy level size zone matrix features (GLSZM), and 50 neighbor gray tone difference matrix features (NGTDM). The intraclass correlation coefficient (ICC) were used to assess intra-observer and inter-observer consistency in ROI feature extraction., with radiomic features having ICCs greater than 0.8 being retained and the features with ICCs less than 0.8 being excluded.

### Radiomics feature selection and establishment of an optimal radiomics model based on machine learning

Firstly, the radiomics features were standardized using the z-score normalization. Subsequently, feature selection was conducted. All signatures were normalized by the z-score method. In the first step, the variance threshold method was applied to select features with a variance of 0.8 or greater, while features with a variance below 0.8 were eliminated. In the second step, Spearman’s correlation analysis was utilized to exclude features with a correlation coefficient exceeding 0.9, thereby reducing multicollinearity between features. Finally, the Least Absolute Shrinkage and Selection Operator (LASSO) regression was used for selecting the optimal radiomics features with non-zero coefficients, and five-fold cross-validation was performed to choose the optimal parameters based on a minimum criterion.

In this study, five machine learning classifiers were used, including Support Vector Machine (SVM), Random Forest (RF), Light Gradient Boosting Machine (LightGBM), Adaptive Boosting (AdaBoost), and Naïve Bayes (NB). The five-fold cross-validation was used to validate the accuracy of the models. The performance of the above models was evaluated using the Receiver Operating Characteristic (ROC) curve and the Area Under the Curve (AUC), along with the calculation of sensitivity, specificity, accuracy, and F1 score.

### Establishment and validation of the clinical model and combined model

**Figure 2:**
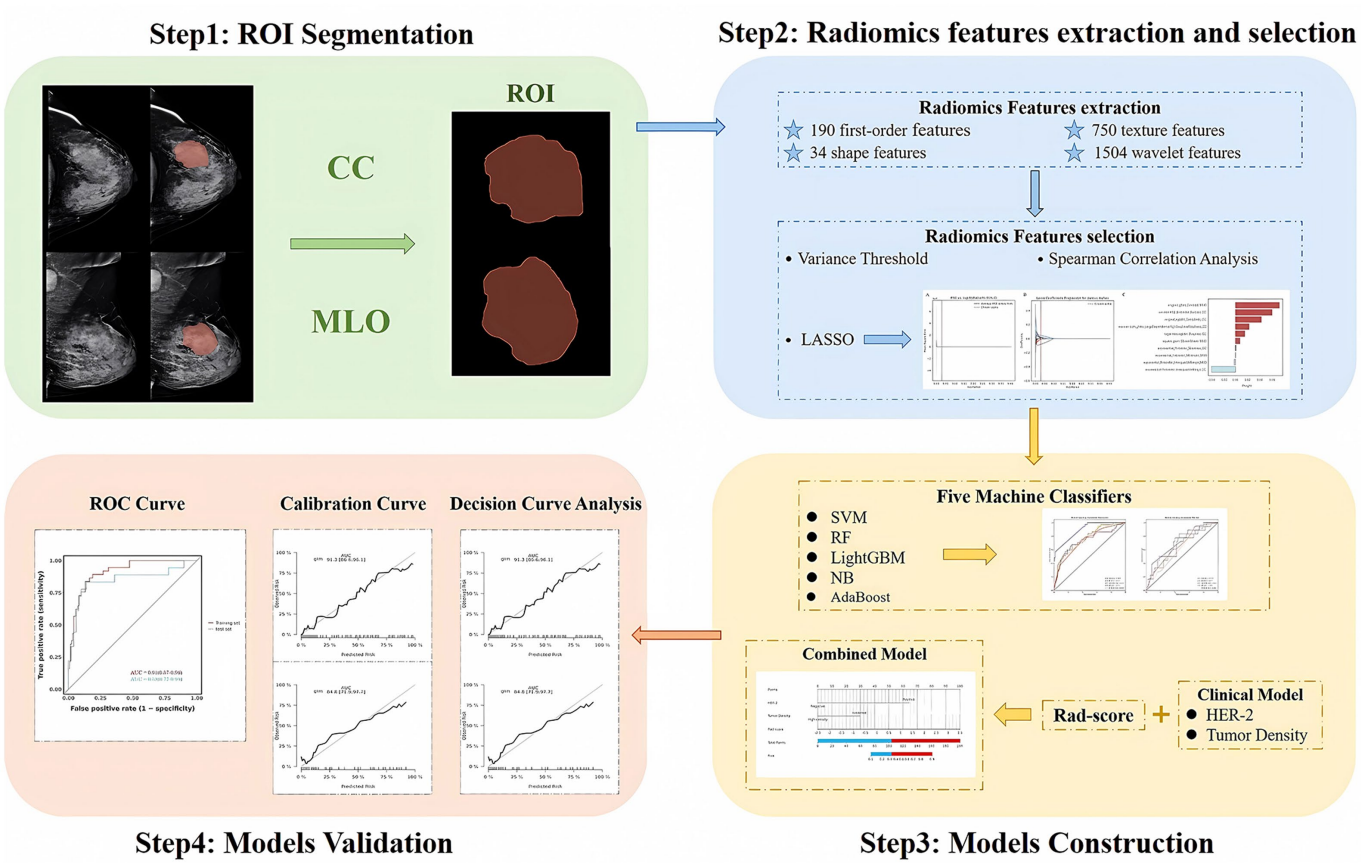
Steps for ROI segmentation, radiomics features extraction and selection, models construction, and validation.

Variables in the training set were screened for independent risk factors associated with the efficacy of NAC in breast cancer using both univariate and multivariate Logistic regression analyses (*p* < 0.05), including clinical-pathological features and FFDM characteristics, to construct a clinical prediction model. The rad-score was calculated using a linear combination of the final selected features weighted by their LASSO coefficients. A combined model was constructed through multivariate logistic regression analysis using the independent risk factors from the clinical model and the rad-score. The performance of each model was assessed using the AUC of the ROC curve. The goodness of fit of the models was evaluated with calibration curves. The decision curve analysis (DCA) was employed to assess the net benefit of radiomics nomogram at various threshold probabilities. The radiomics workflow is shown in Figure 2.

### Probability-based stratification using the nomogram and application of the combined model in an independent cohort of patients

The optimal cutoff value was determined based on the ROC curve of the training set by selecting the value corresponding to the maximum Youden’s index. The optimal cutoff points were used to categorize patients into high-probability and low-probability groups in the nomogram model. Fifty breast cancer patients who underwent NAC were retrospectively identified from Harbin Medical University Cancer Hospital in May 2024. These patients were included as an independent cohort to evaluate the performance of the combined model. The pathological outcomes of these patients were unknown prior to obtaining the predictions from the model.

### Statistical analysis

Radiomics feature extraction and machine learning modeling were performed in Python (v3.7) using the PyRadiomics and scikit-learn libraries. Nomogram construction and statistical validation were implemented in R (v4.2.1) with the rms and pROC packages. The quantitative data were expressed as mean (± standard deviation) or median (interquartile range) [M (P25, P75)], and group comparisons were made using the *t*-test or the Mann–Whitney U test. The categorical data were presented as frequencies (%), and group comparisons were performed using the chi-square test or the Fisher’s exact test. Bilateral *p*-values less than 0.05 were considered statistically significant.

## Results

### Clinical and pathological characteristics

A total of 227 patients were enrolled in this study. The training set included 159 patients (37 PCR, 122 non-PCR), and the test set included 68 patients (18 PCR, 50 non-PCR). There were no statistically significant differences in pathological and radiological characteristics between the training and test sets (*p* > 0.05). In both the training and test sets, the ER status, PR status, HER-2 status, FFDM tumor density, and FFDM tumor margin were significantly associated with PCR (*p* < 0.05). Univariate and multivariate logistic regression analyses identified HER-2 status and FFDM tumor density as independent risk factors for predicting PCR after NAC in breast cancer (Table 1).

**Table 1:** Clinical and pathological characteristics.

Characteristics	Training set (*N* = 159)		*p*-value	Test Set (*N* = 68)		*p*-value
	Non-PCR	PCR		Non-PCR	PCR	
Age, years*	52 ± 10	52 ± 8	0.581	54 ± 9	53 ± 8	0.623
BMI*	25.1 (22.5, 27.1)	24.0 (21.9, 26.8)	0.512	24.65 (23.25, 26.38)	23.58 (22.63, 26.30)	0.341
T stage			0.902			0.760
T1	44 (36%)	14 (38%)		20 (40%)	7 (39%)	
T2	71 (58%)	22 (59%)		27 (54%)	9 (50%)	
T3	7 (6%)	1 (3%)		3 (6%)	2 (11%)	
ER			<0.001			<0.001
Negative	30 (25%)	23 (62%)		13 (26%)	13 (72%)	
Positive	92 (75%)	14 (38%)		37 (74%)	5 (28%)	
PR			<0.001			<0.001
Negative	41 (34%)	28 (76%)		14 (28%)	15 (83%)	
Positive	81 (66%)	9 (24%)		36 (72%)	3 (17%)	
HER-2			<0.001			<0.001
Negative	96 (79%)	7 (19%)		44 (88%)	3 (17%)	
Positive	26 (21%)	30 (81%)		6 (12%)	15 (83%)	
KI-67			0.068			0.160
Low	26 (21%)	3 (8%)		11 (22%)	1 (6%)	
High	96 (79%)	34 (92%)		39 (78%)	17 (94%)	
Breast composition			0.088			0.974
Non-Dense Breast	52 (43%)	10 (27%)		22 (44%)	8 (44%)	
Dense Breast	70 (57%)	27 (73%)		28 (56%)	10 (56%)	
Tumor diameter*	2.81 (2.40, 3.79)	2.60 (2.23, 3.40)	0.130	2.80 (2.43, 3.38)	2.70 (2.53, 3.20)	0.967
Tumor density			<0.001			0.231
Isodense	38 (31%)	23 (62%)		17 (34%)	9 (50%)	
High-density	84 (69%)	14 (38%)		33 (66%)	9 (50%)	
Tumor shape			0.567			>0.999
Round or oval	16 (13%)	3 (8%)		5 (10%)	1 (6%)	
Irregular	106 (87%)	34 (92%)		45 (90%)	17 (94%)	
Tumor Margin			0.037			0.041
Non-spiculated	69 (57%)	28 (76%)		25 (50%)	14 (78%)	
Spiculated	53 (43%)	9 (24%)		25 (50%)	4 (22%)	
Abnormal axillary lymph nodes			0.094			0.322
Negative	60 (49%)	24 (65%)		21 (42%)	10 (56%)	
Positive	62 (51%)	13 (35%)		29 (58%)	8 (44%)	
Calcifications			0.913			0.077
Absent	54 (44%)	16 (43%)		23 (46%)	4 (22%)	
Present	68 (56%)	21 (57%)		27 (54%)	14 (78%)	

*Continuous values expressed as the mean ± standard deviation or median (interquartile range). ER, estrogen receptor; Her2, human epidermal growth factor receptor 2; PR, progesterone receptor; PCR, pathological complete response. Welch Two Sample *t*-test; Wilcoxon rank sum test; Fisher’s exact test; Pearson’s Chi-squared test.

### Radiomics feature selection and establishment of an optimal radiomics model

**Figure 3:**
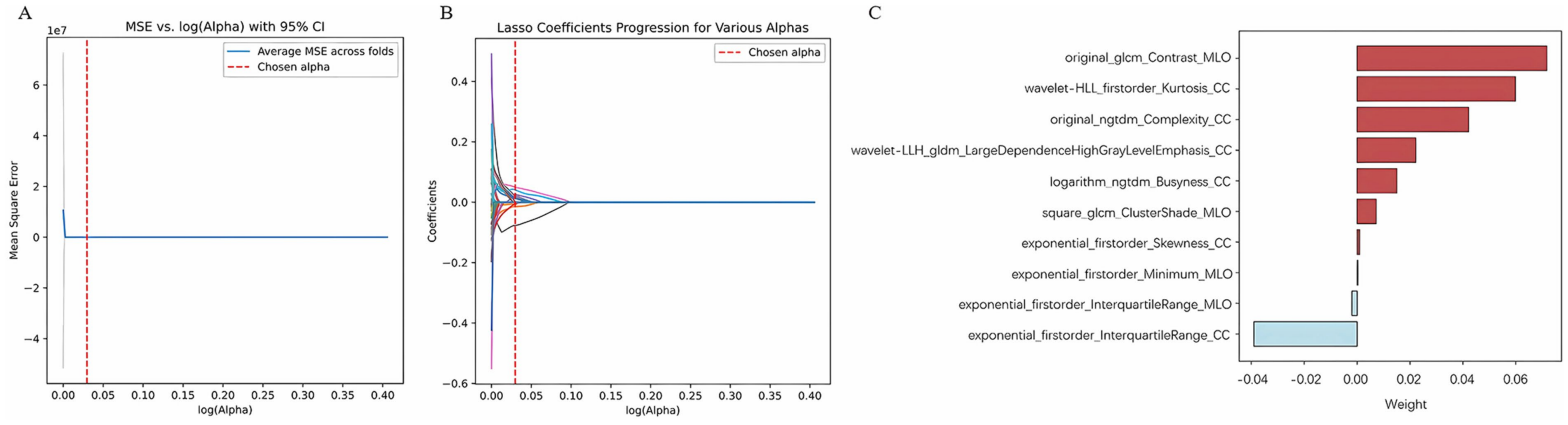
Radiomics features selection by LASSO. **(A)** Using 5-fold cross-validation, select the parameter (*λ*) for the LASSO model based on the minimum criterion; **(B)** LASSO coefficient profiles of the radiomics features; **(C)** Radiomics features.

Through radiomic feature extraction, 2,478 radiomic features were obtained from the FFDM images (both CC and MLO views) of each patient. Among them, 2,254 features with an ICC > 0.8 were retained. A total of 1,072 features were selected using the variance threshold method, 41 features were identified through spearman’s correlation analysis, and ultimately 10 radiomics features were filtered out using the LASSO method (Figure 3). Therefore, after feature selection using the three methods, a total of 10 radiomics features with non-zero coefficients were ultimately retained. Detailed information about the radiomics features can be found in the Supplementary material.

**Figure 4:**
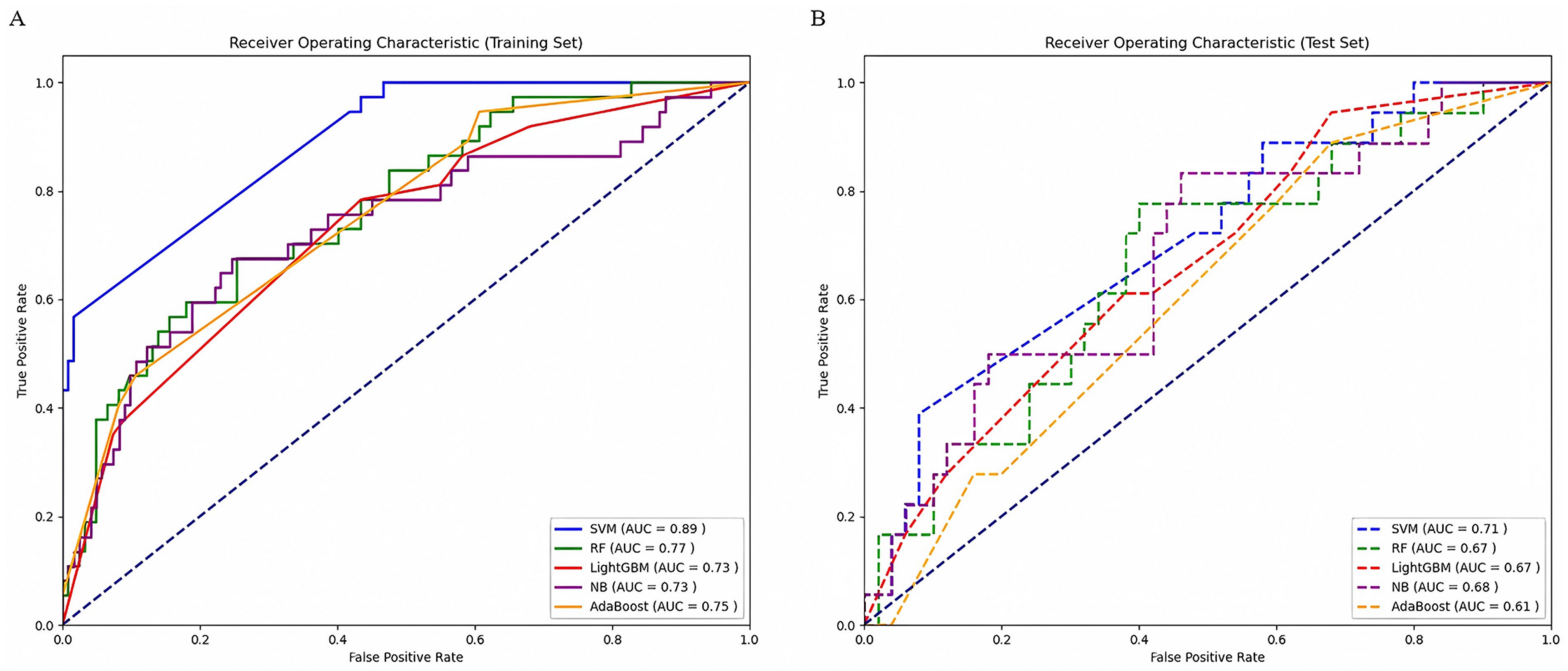
Comparison of ROC curves of five machine learning classifiers in the training set **(A)** and test set **(B)**. AUC, area under the receiver operating characteristic curve; SVM, support vector machine; RF, random forest; LightGBM, Light Gradient Boosting Machine; NB, Naïve Bayes; AdaBoost, Adaptive Boosting.

Ten radiomic features were used to establish a radiomics model based on five machine learning classifiers. Among these, SVM demonstrated the best performance, with a training set AUC of 0.88 (95% CI: 0.84–0.91) and a test set AUC of 0.71 (95% CI: 0.56–0.83) (Figure 4). Table 2 presents the predictive performance of the radiomics models established using the five machine learning classifiers.

**Table 2:** Comparison of diagnostic performance of five machine learning classifiers.

Classifiers	Sets	AUC (95%CI)	Sensitivity	Specificity	Accuracy	F1 Score
SVM	Training	0.89 (0.84–0.94)	0.98	0.58	0.78	0.82
	Test	0.71 (0.58–0.84)	0.72	0.52	0.57	0.47
RF	Training	0.77 (0.68–0.85)	0.81	0.66	0.74	0.76
	Test	0.67 (0.52–0.80)	0.72	0.62	0.65	0.52
LightGBM	Training	0.73 (0.65–0.81)	0.84	0.57	0.7	0.74
	Test	0.67 (0.51–0.80)	0.61	0.62	0.62	0.46
NB	Training	0.73 (0.63–0.83)	0.81	0.62	0.72	0.74
	Test	0.68 (0.53–0.81)	0.78	0.56	0.62	0.52
AdaBoost	Training	0.75 (0.67–0.83)	0.93	0.41	0.67	0.74
	Test	0.61 (0.47–0.74)	0.78	0.4	0.5	0.45

AUC, Area Under the Curve; AdaBoost, Adaptive Boosting; LightGBM, Light Gradient Boosting Machine; NB, Naïve Bayes; RF, random forest; SVM, support vector machine; 95% CI, 95% confidence interval.

### Development and validation of the clinical model and combined model

**Figure 5:**
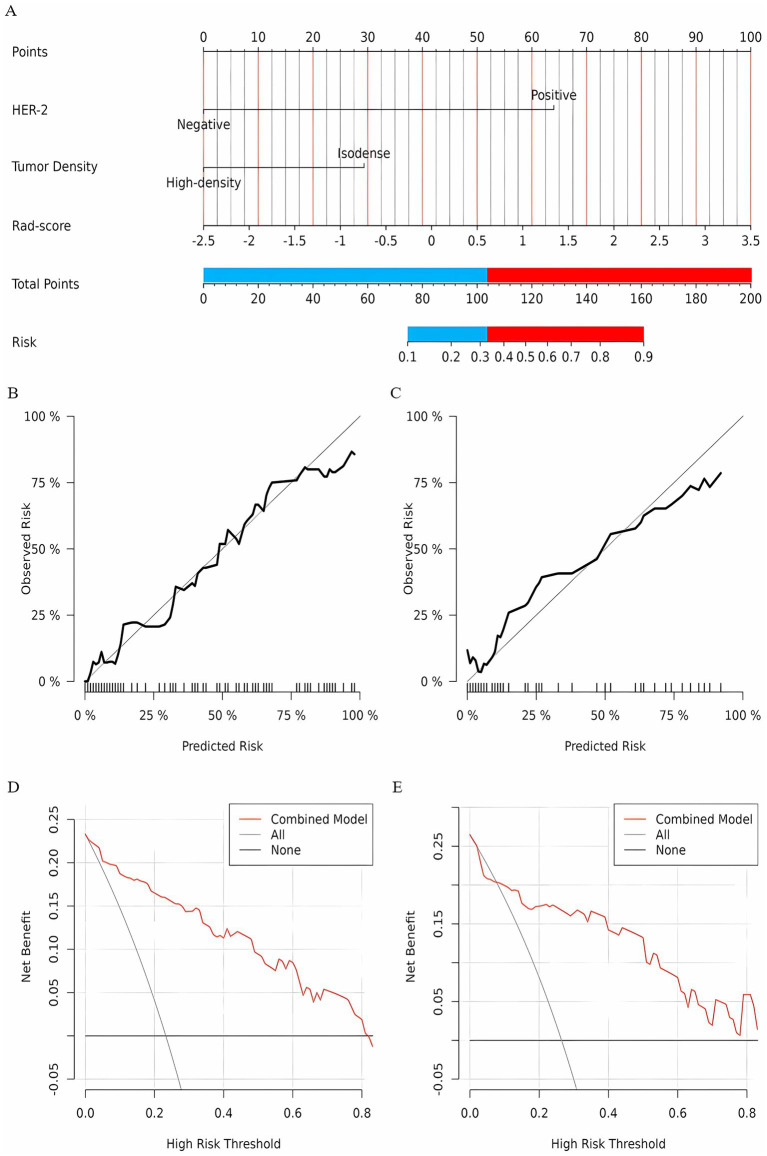
**(A)** A nomogram prediction model for NAC efficacy in breast cancer patients. According to the optimal cutoff points of the red and blue junction, the nomogram is divided into a low-probability group and a high-probability group; The calibration curve of the nomogram prediction model (Panel **B** for the training set, Panel **C** for the test set); Clinical decision curve of the nomogram model (**D** for training set, **E** for test set).

A clinical model was constructed based on independent risk factors, HER-2 and FFDM tumor density. In the training set, the AUC of the clinical model was 0.89 (95% CI: 0.85–0.94); in the test set, the AUC was 0.87 (95% CI: 0.76–0.98). Combining the independent risk factors identified from clinical pathology with the rad-score, a combined model was constructed. Developing a nomogram based on a combined model. In the training set, the combined model achieved an AUC of 0.91 (95% CI: 0.87–0.96), with a sensitivity of 83.8%, specificity of 86.9%, and accuracy of 86.2%. In the test set, the model demonstrated an AUC of 0.85 (95% CI: 0.72–0.98), with a sensitivity of 83.3%, specificity of 86%, and accuracy of 85.3%. The diagnostic performance of the three models is shown in Table 3. The calibration curve of the combined model indicated good model fit, suggesting that the predicted probabilities were consistent with the actual probabilities. The DCA demonstrated that the combined nomogram model yielded good clinical net benefit across a broad range of threshold probabilities in both the training and test sets, indicating favorable clinical performance of the model (Figure 5). The Hosmer-Lemeshow test indicated a good model fit (all *p*-values > 0.05). More information about the calculation of the rad-score can be found in the Supplementary material.

**Table 3:** Diagnostic performance of clinical model, radiomics model, and combined model.

Models	Sets	AUC	Sensitivity	Specificity	Accuracy
Clinical model	Training set	0.89	0.87	0.80	0.82
	Test set	0.87	0.83	0.88	0.87
Radiomics model	Training set	0.88	0.98	0.58	0.78
	Test set	0.71	0.72	0.52	0.57
Combined model	Training set	0.91	0.84	0.87	0.86
	Test set	0.85	0.83	0.86	0.85

AUC, Area Under the Curve.

### Evaluation of the performance of the combined model in an independent cohort

**Figure 6:**
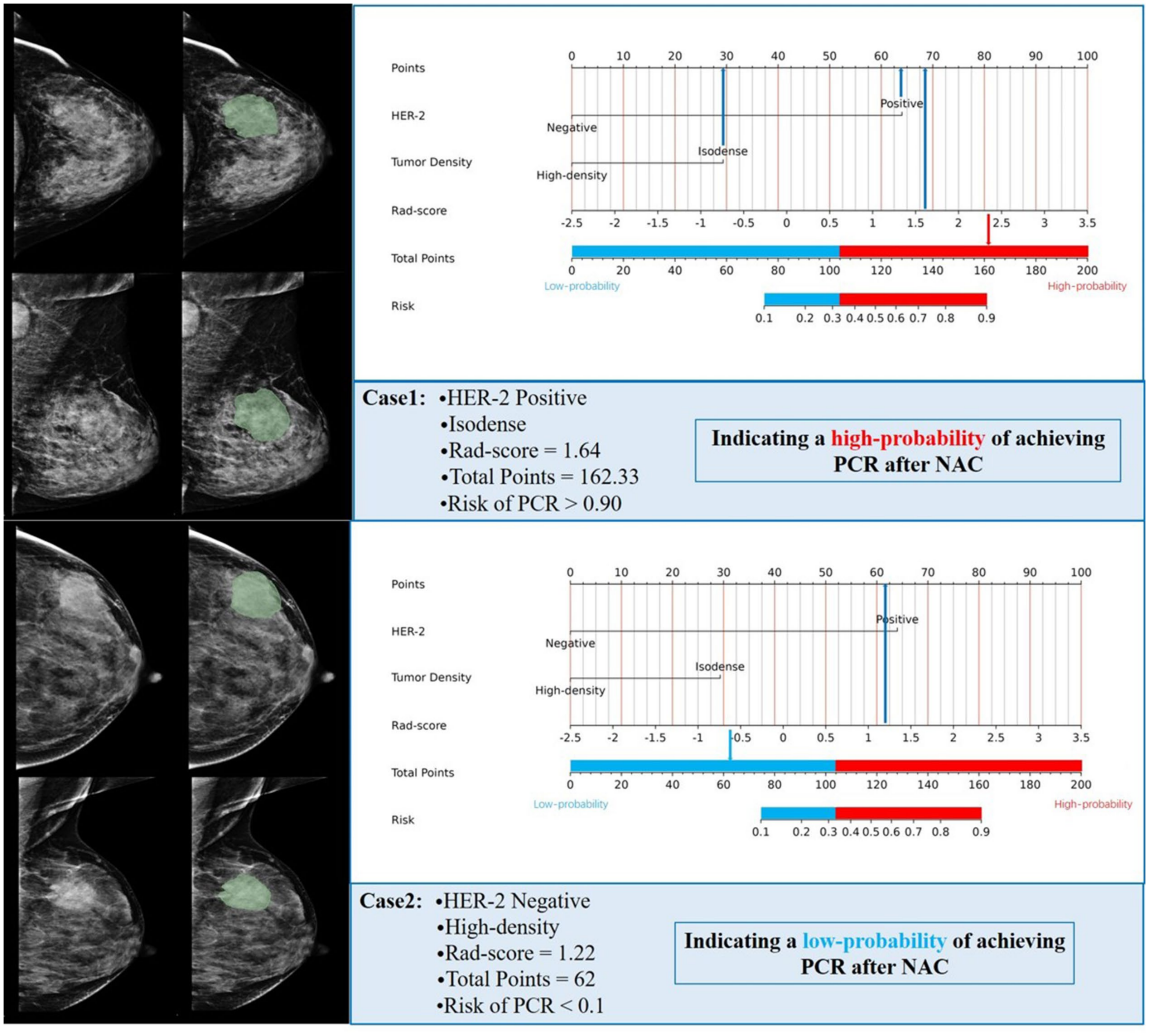
Two examples of using a nomogram model to predict the efficacy of NAC in breast cancer.

Based on the optimal cutoff points, breast cancer patients with a total score ≤ 103.42 were classified into the low-probability group, while those with a total score > 103.42 were classified into the high-probability group. NAC is not recommended for patients in the low-probability group; however, it may be considered for patients in the high-probability group. Of the 50 patients identified at Harbin Medical University Cancer Hospital, 3 did not meet the inclusion and exclusion criteria, resulting in a final cohort of 47 patients included in the study. Rad-score was calculated for these patients, and HER-2 status, FFDM tumor density, and rad-score were incorporated into the combined model. The predicted outcomes for each patient were compared to the optimal cutoff of 103.42 points. The predictive results indicated that 15 patients achieved a PCR after NAC, while 32 patients did not. A comparison of the predictive outcomes with the pathological results revealed that the model erroneously classified one patient who could achieve PCR as unable to do so, and three patients who could not achieve PCR as achieving it. The model demonstrated a sensitivity of 92.3%, specificity of 91.2%, and accuracy of 91.5%. Therefore, we conclude that the nomogram prediction model exhibits stable and accurate predictive performance. Figure 6 presents two examples of using the nomogram model to predict the efficacy of NAC in breast cancer.

## Discussion

NAC, as a crucial therapeutic approach for breast cancer patients, is indicated for those with locally advanced breast cancer, patients requiring tumor downstaging to enable surgery or breast-conserving surgery, individuals with triple-negative or HER2-positive breast cancer, and those at risk of metastasis (4, 22, 23). In this retrospective study, we developed and validated clinical-pathological, radiomics, and combined clinical-radiomics models based on FFDM to predict the therapeutic response of breast cancer patients following NAC. Our results suggest that the clinical-radiomics model holds promise as a decision-making tool for clinicians seeking to provide personalized prediction for patients.

Radiomics can offer a wider selection of higher-order features, enabling a comprehensive and accurate assessment of the relevant information within tumors (24). In this study, we constructed predictive models based on FFDM radiomics features using five machine learning classifiers. The SVM classifier demonstrated high and stable predictive performance in both the training and test sets, with AUC values of 0.88 and 0.71, respectively. SVM is particularly powerful at identifying subtle patterns in complex datasets and can achieve optimal generalization with limited sample information. It offers advantages such as high accuracy and low computational demand (25, 26). Additionally, the SVM algorithm is commonly used in classification and prediction methods and demonstrates high accuracy (27). The study by Zhu et al. (28) developed five machine learning models based on DCE-MRI radiomic features to predict sentinel lymph node metastasis in breast cancer. The results indicated that the SVM model achieved the highest AUC of 0.86 in the validation set, demonstrating the best predictive performance. This is similar to our research findings (28). Zhang et al. (29) developed radiomic, pathological, deep learning pathological, and deep learning radiopathomics (DLRPM) models using the SVM method to predict PCR in breast cancer patients. The results showed that in the training set, the DLRPM model outperformed the other three single-scale prediction models in terms of AUC, sensitivity, specificity, accuracy, and other metrics.

In this study, the clinical-pathological model achieved an AUC of 0.89 in the training set, surpassed most existing models (30, 31). Univariate and multivariate logistic regression analyses identified HER2 status and FFDM tumor density as independent risk factors for predicting the efficacy of NAC in breast cancer. Both factors demonstrated statistical significance (*p* < 0.05). This study showed that HER-2 positive breast cancer patients were more likely to achieve a PCR after NAC, which is consistent with previous reports (32, 33). NAC is clearly indicated for HER-2 positive patients, likely due to the addition of HER-2 targeted therapies (e.g., trastuzumab and pertuzumab) during treatment. HER-2 positive breast cancer patients are particularly responsive to these agents, resulting in higher PCR rates following NAC (34, 35). In addition, the results of this study showed that breast cancer patients with isodense tumors on FFDM were more likely to achieve a PCR after NAC compared to those with high-density tumors. In FFDM examination, the density of a mass refers to the result of comparing its density to that of normal breast tissue of the same volume in the surrounding or contralateral breast. High-density masses in breast cancer patients are the most common direct X-ray signs, which may be attributed to factors such as the dense arrangement of cancer cells, higher mineral content, increased blood vessels, and fibroplasia. Compared to low- or isodense masses, high-density masses may require higher pressure gradients and drug dosages to achieve the same therapeutic effect. Therefore, breast cancer patients with high-density masses are less likely to achieve a PCR after NAC (36, 37).

To improve the accuracy of the predictive model, we developed a combined model incorporating the rad-score and two independent clinical risk factors. This model demonstrated a significant advantage in predicting the efficacy of NAC in breast cancer. A nomogram was developed based on this combined model, with an AUC of 0.91 for the training set and 0.85 for the test set. Chen et al. (38) developed a radiomics nomogram model using MRI images to predict the efficacy of NAC in breast cancer. The AUC for the training set was 0.89, and for the test set, it was 0.88, both significantly higher than that of the radiomics signature. These results suggest that the model can effectively assist clinicians in predicting breast cancer patients’ responses to NAC, aligning with our findings. Liu et al. (31) established radiomic models based on ultrasound image features before (RS1) and after two cycles of NAC (RS2), and developed a nomogram model combining clinicopathological, and ultrasound features to predict NAC efficacy. The results showed that the AUC for RS2 was higher than that for RS1 and Delta-RS/RS1. The nomogram model combining RS2 with clinicopathological, and ultrasound features demonstrated superior AUCs in both the training set and test set compared to RS2 alone (0.90 vs. 0.86, 0.89 vs. 0.82). Furthermore, we determined that the optimal cutoff points for the nomogram are 103.42. Patients with a total score greater than 103.42 are more likely to achieve PCR after NAC. For this high-probability group, clinicians may consider recommending NAC (39). However, it should be noted that the optimal cutoff value (103.42) determined in this study may be data-dependent. The optimization of the Youden index relies on the distribution characteristics of the training set. Although external validation shows that the model remains robust, the cutoff value may need to be dynamically adjusted in other medical institutions or populations (e.g., different races, treatment protocols). It is important to emphasize that the core value of the nomogram lies in integrating multidimensional predictive factors to provide individualized PCR probability predictions, while the cutoff value is merely a tool for converting continuous probabilities into binary classification. Clinicians can freely adjust the threshold based on practical needs.

In recent years, nomogram models have been widely applied in clinical practice due to their intuitive nature, personalized predictions, and ease of use without the need for complex calculations (40). In this study, clinicians can obtain individualized predictions of PCR probability by simply inputting HER-2 status, FFDM tumor density, and rad-score into the nomogram model. However, it should be noted that the calculation of rad-score currently requires manual delineation of the tumor ROI by radiologists, which may impose additional workflow burdens. Future research will focus on developing deep learning-based automated ROI segmentation algorithms to streamline this process and enhance clinical utility.

This study had certain limitations: First, as a single-center retrospective study, the homogeneity of the patient population and the inclusion criteria may introduce selection bias. Although external validation indicates the model’s robust performance, variations in imaging equipment parameters and treatment strategies across different medical centers could impact its practical application. Therefore, future multi-center prospective studies are required to further assess the generalizability of the model. Second, the study included only mass-forming breast cancer, and the efficacy of NAC in non-mass-forming breast cancer remained to be further investigated. Third, as a retrospective study, there was a potential for selection bias, which might have reduced the reliability of the predictive. Model. Fourth, the sample size in this study was relatively small; future research will involve increasing the sample size to further validate the findings. Fifth, although double-blind manual segmentation and ICC-based feature selection ensured consistency, observer bias remains a potential limitation. Future studies will implement deep learning-based automated segmentation to reduce variability and enhance reproducibility. Sixth, while non-linear models may capture complex feature interactions, we prioritized linear methods (LASSO regression) to ensure clinical interpretability and prevent overfitting in our limited sample, with systematic comparisons planned in future work.

## Conclusion

In conclusion, the nomogram developed by integrating clinical pathological features and rad-score demonstrated superior predictive performance, with probability-based stratification enhancing the clarity and specificity of the results. This non-invasive preoperative prediction method can provide personalized treatment decision-making guidance for clinicians.

## Data Availability

The datasets presented in this article are not readily available because the dataset is restricted due to privacy concerns, and patient information has been de-identified in accordance with ethical guidelines. Requests to access the datasets should be directed to YR, ruanye2000@163.com.
