# Supplementary material for: Personalized predictions of neoadjuvant chemotherapy response in breast cancer using machine learning and full-field digital mammography radiomics
Source: Front Med (Lausanne). 2025 Apr 17;12:1582560. doi: 10.3389/fmed.2025.1582560 (PMC12043669; doi:10.3389/fmed.2025.1582560)
Supplement: Supplementary file 1 [file Data_Sheet_1.docx]

**Personalized Predictions of Neoadjuvant Chemotherapy Response in Breast Cancer Using Machine Learning and Full-field Digital Mammography Radiomics**

1. **Radiomics feature selection**

Our study identified 10 non-zero coefficient radiomics features through variance thresholding, Spearman analysis, and LASSO selection. These features are: exponential_firstorder_InterquartileRange_CC; exponential_firstorder_Skewness_CC; logarithm_ngtdm_Busyness_CC; original_ngtdm_Complexity_CC; wavelet-LLH_gldm_LargeDependenceHighGrayLevelEmphasis_CC; wavelet-HLL_firstorder_Kurtosis_CC; exponential_firstorder_InterquartileRange_MLO; exponential_firstorder_Minimum_MLO; original_glcm_Contrast_MLO; and square_glcm_ClusterShade_MLO. The coefficients corresponding to these ten features are shown in Table S1.

**Table S1** Radiomics features and their coefficients.

| **Radiomics features** | **Coefficient** |
| --- | --- |
| Exponential_firstorder_InterquartileRange_CC | -0.039049317 |
| Exponential_firstorder_Skewness_CC | 0.00098 |
| logarithm_ngtdm_Busyness_CC | 0.015001 |
| logarithm_ngtdm_Busyness_CC | 0.042159 |
| Wavelet-LLH_gldm_LargeDependenceHighGrayLevelEmphasis_CC | 0.022184 |
| Wavelet-HLL_firstorder_Kurtosis_CC | 0.059844 |
| Exponential_firstorder_InterquartileRange_MLO | -0.00197 |
| Exponential_firstorder_Minimum_MLO | 0.000334 |
| Original_glcm_Contrast_MLO | 0.071736 |
| Square_glcm_ClusterShade_MLO | 0.007209 |

1. **Rad-score calculation**

The rad-score was calculated using the following formula: Rad-score=

-0.039049317 * exponential_firstorder_InterquartileRange_CC

+ 0.000980226 * exponential_firstorder_Skewness_CC

+ 0.015001232 * logarithm_ngtdm_Busyness_CC

+ 0.042159236 * original_ngtdm_Complexity_CC

+ 0.02218408 * wavelet-LLH_gldm_LargeDependenceHighGrayLevelEmphasis_CC

+ 0.059844 * wavelet-HLL_firstorder_Kurtosis_CC

- 0.001965431 * exponential_firstorder_InterquartileRange_MLO

+ 0.000333915 * exponential_firstorder_Minimum_MLO

+ 0.071735983 * original_glcm_Contrast_MLO

+ 0.007209362 * square_glcm_ClusterShade_MLO.
